# *catena*-Poly[[tetra­aqua­zinc(II)]-μ-1,3,4-thia­diazol-2,5-diyldithio­diacetato-κ^2^ *O*:*O*′]

**DOI:** 10.1107/S1600536808013251

**Published:** 2008-05-10

**Authors:** Ying-Hui Yu, Chuan He, Guang-Feng Hou, Jin-Sheng Gao, Hong-Kun Zhang

**Affiliations:** a College of Chemistry and Materials Science, Heilongjiang University, Harbin 150080, People’s Republic of China; b School of Resources and Safety Engineering, China University of Mining and Techology (Beijing Campus), Beijing 100083, People’s Republic of China; c Department of Food and Environmental Engineering, Heilongjiang East College, Harbin 150086, People’s Republic of China

## Abstract

In the title linear coordination polymer, [Zn(C_6_H_4_N_2_O_4_S_3_)(H_2_O)_4_]_*n*_, the Zn^II^ atom is coordinated by four O atoms from four water mol­ecules and two O atoms from two [5-(carb­oxyl­atomethyl­sulfan­yl)-1,3,4-thia­diazol-2-ylsulfan­yl]acetate units in an octa­hedral coordination environment. The chains are linked into a three-dimensional supra­molecular network *via* O—H⋯O and O—H⋯N hydrogen bonds.

## Related literature

For the structure of other metal 1,3,4-thia­diazolyl-2,5-dithio­acetates, see Gao *et al.* (2005 ▶, 2006 ▶); Zhang *et al.* (2006 ▶).
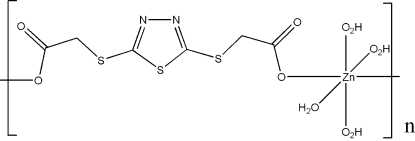


## Experimental

### 

#### Crystal data

[Zn(C_6_H_4_N_2_O_4_S_3_)(H_2_O)_4_]
*M* *_r_* = 401.73
Monoclinic, 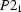

*a* = 5.1554 (10) Å
*b* = 9.5043 (19) Å
*c* = 13.627 (3) Å
β = 94.82 (3)°
*V* = 665.3 (2) Å^3^
*Z* = 2
Mo *K*α radiation
μ = 2.35 mm^−1^
*T* = 291 (2) K
0.42 × 0.18 × 0.18 mm

#### Data collection

Rigaku R-AXIS RAPID diffractometer
Absorption correction: multi-scan (*ABSCOR*; Higashi, 1995 ▶) *T*_min_ = 0.439, *T*_max_ = 0.675
6408 measured reflections
2774 independent reflections
2624 reflections with *I* > 2σ(*I*)
*R*_int_ = 0.022

#### Refinement

*R*[*F* ^2^ > 2σ(*F* ^2^)] = 0.021
*wR*(*F* ^2^) = 0.046
*S* = 1.06
2774 reflections
181 parameters
1 restraint
H-atom parameters constrained
Δρ_max_ = 0.26 e Å^−3^
Δρ_min_ = −0.33 e Å^−3^
Absolute structure: Flack (1983 ▶), 1151 Friedel pairs
Flack parameter: 0.014 (8)

### 

Data collection: *RAPID-AUTO* (Rigaku, 1998 ▶); cell refinement: *RAPID-AUTO*; data reduction: *CrystalStructure* (Rigaku/MSC, 2002 ▶); program(s) used to solve structure: *SHELXS97* (Sheldrick, 2008 ▶); program(s) used to refine structure: *SHELXL97* (Sheldrick, 2008 ▶); molecular graphics: *SHELXTL* (Sheldrick, 2008 ▶); software used to prepare material for publication: *SHELXL97*.

## Supplementary Material

Crystal structure: contains datablocks global, I. DOI: 10.1107/S1600536808013251/ng2446sup1.cif

Structure factors: contains datablocks I. DOI: 10.1107/S1600536808013251/ng2446Isup2.hkl

Additional supplementary materials:  crystallographic information; 3D view; checkCIF report

## Figures and Tables

**Table 1:** Hydrogen-bond geometry (Å, °)

*D*—H⋯*A*	*D*—H	H⋯*A*	*D*⋯*A*	*D*—H⋯*A*
O5—H6⋯O3	0.85	2.53	2.971 (3)	113
O5—H6⋯O6^i^	0.85	2.23	3.053 (3)	165
O5—H5⋯N2^i^	0.85	2.05	2.897 (3)	172
O6—H8⋯O4^ii^	0.85	2.02	2.762 (2)	145
O6—H7⋯O7^ii^	0.85	2.36	3.116 (3)	148
O7—H10⋯O3^iii^	0.85	1.94	2.770 (3)	166
O7—H9⋯O1^iv^	0.85	2.61	2.992 (2)	109
O7—H9⋯O2^iv^	0.85	1.85	2.680 (3)	166
O8—H12⋯N1^i^	0.85	1.98	2.819 (3)	172
O8—H11⋯O1^v^	0.85	1.87	2.713 (2)	175

Symmetry codes: (i) 
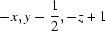
; (ii) 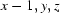; (iii) 
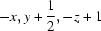
; (iv) 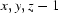; (v) 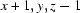.

**Fig. 1.**
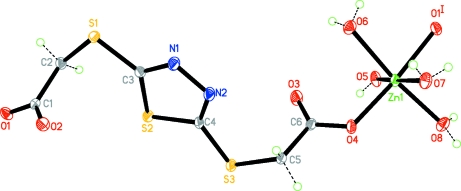
The molecular structure of (I), showing displacement ellipsoids at the 30% probability level for non-H atoms.

**Fig. 2.**
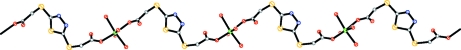
One-dimensional chain structure of the title complex. H atoms have been omitted for clarity.

**Fig. 3.**
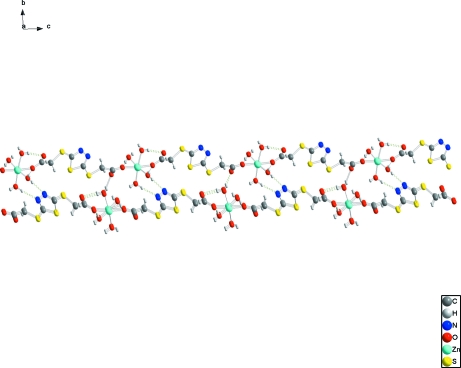
A partial packing view, showing the three-dimensional hydrogen-bonding network. Dashed lines indicate the hydrogen-bonding interactions. H atoms not involved in hydrogen bonds have been omitted for clarity.

## supplementary crystallographic information

### Comment

1,3,4-Thiadiazolyl-2,5-dithioacetic acid is a multidentate flexible aromatic carboxylic acid having two —S—CH_2_CO_2_H arms, and its N atoms can be considered as a potential coordinate candidate which also would coordinate with metal atoms fomed a supramolecular complexes. The structure of 1,3,4-Thiadiazolyl-2,5-dithioacetic acid was reported by Gao *et al.* (2005) and Zhang *et al.*, (2006). In this paper, we reporte a new one-dimensional title compound crystal structure, synthesized by the reaction of 1,3,4-Thiadiazolyl-2,5-dithioacetic acid and zinc dichloride in aqueous solution.

Complex (I) consists of molecules of tetraaquazinc(II)-1,3,4-thiadiazol-2,5-diyldithiodiacetato. The zinc atom is six-coordinated in an octahedron environment (Figure 1), each zinc atom connect with two 1,3,4-Thiadiazolyl-2,5-dithioacetic acid ligand and four water molecules formed a one-dimensional chain structure along *c* axis (Figure 2).

There are eight symmetry-independent 'active' H atoms in the crystal structure; all of them participate in hydrogen bonds, which link the one-dimensional chain structure into an infinite three-dimensional network (Table 1, Figure 3).

### Experimental

1,3,4-Thiadiazolyl-2,5-dithioacetic acid was prepared from 2,5-dimercapto-1,3,4-thiadiazole, using the method for synthesis of benzene-1,2-dioxyacetic acid reported by us (Gao *et al.*, 2006). The colorless zinc complex was obtained from the reaction of zinc dichloride hexahydrate (0.244 g, 1 mmol) and 1,3,4-Thiadiazolyl-2,5-dithioacetic acid (0.532 g, 2 mmol) in hot water (20 ml), and then the pH was adjusted to about 6 with 0.2 *M* sodium hydroxide. The resulting solution was filtered and allowed to stand in a desiccator at room temperature for several days.

### Refinement

H atoms bound to C atoms were placed in calculated positions and treated as riding on their parent atoms, with C—H = 0.97 Å (methylene) and with *U*_iso_(H) = 1.2*U*_eq_(C). Water H atoms were initially located in a difference Fourier map but they were treated as riding on their parent atoms with O—H = 0.85 Å, and with *U*_iso_(H) = 1.5*U*_eq_(O).

### Crystal data

**Table:**

[Zn(C_6_H_4_N_2_O_4_S_3_)(H_2_O)_4_]	*F*_000_ = 408
*M**_r_* = 401.73	*D*_x_ = 2.005 Mg m^−^^3^
Monoclinic, *P*2_1_	Mo *K*α radiation λ = 0.71073 Å
Hall symbol: P 2yb	Cell parameters from 6179 reflections
*a* = 5.1554 (10) Å	θ = 3.0–27.5º
*b* = 9.5043 (19) Å	µ = 2.35 mm^−^^1^
*c* = 13.627 (3) Å	*T* = 291 (2) K
β = 94.82 (3)º	Block, colorless
*V* = 665.3 (2) Å^3^	0.42 × 0.18 × 0.18 mm
*Z* = 2	

### Data collection

**Table:**

Rigaku R-AXIS RAPID diffractometer	2774 independent reflections
Radiation source: fine-focus sealed tube	2624 reflections with *I* > 2σ(*I*)
Monochromator: graphite	*R*_int_ = 0.022
*T* = 291(2) K	θ_max_ = 27.5º
ω scans	θ_min_ = 3.0º
Absorption correction: multi-scan(ABSCOR; Higashi, 1995)	*h* = −6→6
*T*_min_ = 0.439, *T*_max_ = 0.675	*k* = −11→12
6408 measured reflections	*l* = −17→17

### Refinement

**Table:**

Refinement on *F*^2^	Hydrogen site location: inferred from neighbouring sites
Least-squares matrix: full	H-atom parameters constrained
*R*[*F*^2^ > 2σ(*F*^2^)] = 0.021	*w* = 1/[σ^2^(*F*_o_^2^) + (0.0189*P*)^2^] where *P* = (*F*_o_^2^ + 2*F*_c_^2^)/3
*wR*(*F*^2^) = 0.046	(Δ/σ)_max_ = 0.001
*S* = 1.06	Δρ_max_ = 0.26 e Å^−^^3^
2774 reflections	Δρ_min_ = −0.32 e Å^−^^3^
181 parameters	Extinction correction: none
1 restraint	Absolute structure: Flack (1983), 1151 Friedel pairs
Primary atom site location: structure-invariant direct methods	Flack parameter: 0.014 (8)
Secondary atom site location: difference Fourier map	

### Special details

**Table:**

Geometry. All e.s.d.'s (except the e.s.d. in the dihedral angle between two l.s. planes) are estimated using the full covariance matrix. The cell e.s.d.'s are taken into account individually in the estimation of e.s.d.'s in distances, angles and torsion angles; correlations between e.s.d.'s in cell parameters are only used when they are defined by crystal symmetry. An approximate (isotropic) treatment of cell e.s.d.'s is used for estimating e.s.d.'s involving l.s. planes.
Refinement. Refinement of *F*^2^ against ALL reflections. The weighted *R*-factor *wR* and goodness of fit *S* are based on *F*^2^, conventional *R*-factors *R* are based on *F*, with *F* set to zero for negative *F*^2^. The threshold expression of *F*^2^ > σ(*F*^2^) is used only for calculating *R*-factors(gt) *etc*. and is not relevant to the choice of reflections for refinement. *R*-factors based on *F*^2^ are statistically about twice as large as those based on *F*, and *R*- factors based on ALL data will be even larger.

### Fractional atomic coordinates and isotropic or equivalent isotropic displacement parameters (Å^2^)

**Table:**

	*x*	*y*	*z*	*U*_iso_*/*U*_eq_	
C1	−0.1447 (4)	0.4252 (3)	1.17156 (18)	0.0201 (5)	
C2	−0.3790 (4)	0.4114 (3)	1.09577 (18)	0.0218 (5)	
H1	−0.5359	0.4172	1.1301	0.026*	
H2	−0.3749	0.3184	1.0665	0.026*	
C3	−0.1709 (4)	0.4813 (2)	0.92174 (18)	0.0199 (5)	
C4	0.1357 (4)	0.3689 (2)	0.82727 (18)	0.0213 (5)	
C5	0.4372 (4)	0.3491 (3)	0.67199 (16)	0.0211 (4)	
H3	0.4337	0.4499	0.6833	0.025*	
H4	0.6113	0.3248	0.6558	0.025*	
C6	0.2476 (4)	0.3147 (2)	0.58475 (18)	0.0207 (5)	
N1	−0.1311 (4)	0.5509 (2)	0.84268 (16)	0.0278 (5)	
N2	0.0486 (4)	0.4848 (2)	0.78684 (16)	0.0280 (5)	
O1	−0.1686 (3)	0.3552 (2)	1.24944 (12)	0.0262 (4)	
O2	0.0419 (3)	0.5006 (2)	1.15424 (13)	0.0272 (4)	
O3	0.0336 (3)	0.2622 (2)	0.59581 (14)	0.0328 (4)	
O4	0.3282 (3)	0.3463 (3)	0.50187 (11)	0.0275 (3)	
O5	−0.0626 (3)	0.14243 (19)	0.39464 (14)	0.0290 (4)	
H5	−0.0598	0.0891	0.3447	0.044*	
H6	0.0049	0.1028	0.4466	0.044*	
O6	−0.2103 (3)	0.45593 (19)	0.44603 (14)	0.0279 (4)	
H7	−0.3137	0.5040	0.4074	0.042*	
H8	−0.3032	0.3988	0.4759	0.042*	
O7	0.2564 (3)	0.54165 (19)	0.33802 (14)	0.0274 (4)	
H9	0.2034	0.5402	0.2772	0.041*	
H10	0.1894	0.6112	0.3658	0.041*	
O8	0.3690 (3)	0.2453 (2)	0.29289 (14)	0.0291 (4)	
H11	0.5185	0.2761	0.2817	0.044*	
H12	0.3081	0.1896	0.2479	0.044*	
S1	−0.40220 (11)	0.53897 (6)	0.99806 (5)	0.02380 (13)	
S2	0.00595 (10)	0.32704 (7)	0.93689 (4)	0.02359 (12)	
S3	0.37405 (12)	0.26074 (7)	0.78411 (5)	0.02603 (14)	
Zn1	0.09545 (4)	0.34105 (3)	0.374965 (19)	0.02112 (7)	

### Atomic displacement parameters (Å^2^)

**Table:**

	*U*^11^	*U*^22^	*U*^33^	*U*^12^	*U*^13^	*U*^23^
C1	0.0183 (10)	0.0232 (12)	0.0194 (14)	0.0027 (9)	0.0046 (9)	−0.0024 (10)
C2	0.0213 (10)	0.0259 (12)	0.0183 (13)	−0.0012 (9)	0.0025 (9)	0.0012 (10)
C3	0.0216 (11)	0.0202 (12)	0.0177 (13)	0.0025 (9)	0.0008 (8)	−0.0002 (9)
C4	0.0243 (10)	0.0253 (14)	0.0143 (12)	0.0034 (9)	0.0026 (8)	0.0016 (9)
C5	0.0212 (9)	0.0246 (11)	0.0180 (11)	0.0037 (11)	0.0039 (8)	0.0002 (12)
C6	0.0219 (10)	0.0218 (13)	0.0187 (12)	0.0012 (9)	0.0043 (8)	−0.0012 (9)
N1	0.0349 (11)	0.0273 (11)	0.0222 (12)	0.0108 (9)	0.0086 (9)	0.0074 (9)
N2	0.0352 (11)	0.0306 (12)	0.0193 (12)	0.0100 (9)	0.0089 (9)	0.0079 (9)
O1	0.0229 (7)	0.0355 (10)	0.0199 (9)	−0.0069 (8)	−0.0007 (6)	0.0066 (9)
O2	0.0226 (8)	0.0360 (10)	0.0233 (10)	−0.0072 (7)	0.0047 (7)	0.0047 (8)
O3	0.0296 (9)	0.0432 (11)	0.0261 (11)	−0.0132 (8)	0.0053 (8)	0.0036 (9)
O4	0.0249 (7)	0.0422 (9)	0.0159 (8)	−0.0072 (9)	0.0044 (6)	−0.0006 (10)
O5	0.0394 (10)	0.0293 (9)	0.0182 (10)	−0.0110 (8)	0.0016 (7)	−0.0012 (8)
O6	0.0249 (8)	0.0308 (10)	0.0291 (11)	−0.0037 (7)	0.0088 (7)	0.0034 (8)
O7	0.0337 (9)	0.0244 (9)	0.0250 (10)	−0.0053 (7)	0.0079 (7)	−0.0032 (8)
O8	0.0223 (8)	0.0364 (11)	0.0296 (11)	−0.0093 (7)	0.0075 (7)	−0.0132 (8)
S1	0.0246 (3)	0.0267 (3)	0.0207 (3)	0.0070 (2)	0.0053 (2)	0.0032 (2)
S2	0.0284 (2)	0.0245 (3)	0.0185 (3)	0.0081 (3)	0.0058 (2)	0.0065 (3)
S3	0.0302 (3)	0.0303 (3)	0.0183 (3)	0.0106 (3)	0.0058 (2)	0.0043 (3)
Zn1	0.02055 (11)	0.02492 (14)	0.01799 (14)	−0.00523 (11)	0.00214 (9)	−0.00268 (13)

### Geometric parameters (Å, °)

**Table:**

C1—O2	1.238 (3)	C6—O4	1.272 (3)
C1—O1	1.267 (3)	N1—N2	1.397 (3)
C1—C2	1.527 (3)	O1—Zn1^i^	2.0989 (17)
C2—S1	1.797 (2)	O4—Zn1	2.0209 (17)
C2—H1	0.9700	O5—Zn1	2.0824 (18)
C2—H2	0.9700	O5—H5	0.8500
C3—N1	1.295 (3)	O5—H6	0.8500
C3—S2	1.730 (2)	O6—Zn1	2.2072 (17)
C3—S1	1.736 (2)	O6—H7	0.8500
C4—N2	1.295 (3)	O6—H8	0.8500
C4—S2	1.734 (2)	O7—Zn1	2.1557 (18)
C4—S3	1.742 (2)	O7—H9	0.8501
C5—C6	1.510 (3)	O7—H10	0.8500
C5—S3	1.797 (2)	O8—Zn1	2.0815 (17)
C5—H3	0.9700	O8—H11	0.8500
C5—H4	0.9700	O8—H12	0.8500
C6—O3	1.231 (3)	Zn1—O1^ii^	2.0989 (17)
			
O2—C1—O1	126.5 (2)	Zn1—O5—H6	111.8
O2—C1—C2	120.2 (2)	H5—O5—H6	111.7
O1—C1—C2	113.2 (2)	Zn1—O6—H7	115.4
C1—C2—S1	116.27 (17)	Zn1—O6—H8	110.2
C1—C2—H1	108.2	H7—O6—H8	106.9
S1—C2—H1	108.2	Zn1—O7—H9	96.6
C1—C2—H2	108.2	Zn1—O7—H10	113.9
S1—C2—H2	108.2	H9—O7—H10	109.7
H1—C2—H2	107.4	Zn1—O8—H11	127.7
N1—C3—S2	114.44 (18)	Zn1—O8—H12	115.7
N1—C3—S1	120.11 (17)	H11—O8—H12	111.7
S2—C3—S1	125.31 (14)	C3—S1—C2	102.97 (11)
N2—C4—S2	114.60 (17)	C3—S2—C4	86.59 (11)
N2—C4—S3	125.98 (19)	C4—S3—C5	101.23 (12)
S2—C4—S3	119.35 (13)	O4—Zn1—O8	95.17 (7)
C6—C5—S3	114.60 (17)	O4—Zn1—O5	97.06 (8)
C6—C5—H3	108.6	O8—Zn1—O5	87.88 (7)
S3—C5—H3	108.6	O4—Zn1—O1^ii^	173.48 (9)
C6—C5—H4	108.6	O8—Zn1—O1^ii^	90.71 (7)
S3—C5—H4	108.6	O5—Zn1—O1^ii^	85.94 (7)
H3—C5—H4	107.6	O4—Zn1—O7	88.02 (8)
O3—C6—O4	124.6 (2)	O8—Zn1—O7	88.26 (7)
O3—C6—C5	121.2 (2)	O5—Zn1—O7	173.88 (8)
O4—C6—C5	114.17 (19)	O1^ii^—Zn1—O7	89.36 (7)
C3—N1—N2	112.4 (2)	O4—Zn1—O6	90.40 (7)
C4—N2—N1	111.9 (2)	O8—Zn1—O6	173.27 (8)
C1—O1—Zn1^i^	127.90 (15)	O5—Zn1—O6	95.18 (7)
C6—O4—Zn1	122.62 (14)	O1^ii^—Zn1—O6	83.55 (7)
Zn1—O5—H5	113.8	O7—Zn1—O6	88.17 (6)

Symmetry codes: (i) *x*, *y*, *z*+1; (ii) *x*, *y*, *z*−1.

### Hydrogen-bond geometry (Å, °)

**Table:**

*D*—H···*A*	*D*—H	H···*A*	*D*···*A*	*D*—H···*A*
O5—H6···O3	0.85	2.53	2.971 (3)	113
O5—H6···O6^iii^	0.85	2.23	3.053 (3)	165
O5—H5···N2^iii^	0.85	2.05	2.897 (3)	172
O6—H8···O4^iv^	0.85	2.02	2.762 (2)	145
O6—H7···O7^iv^	0.85	2.36	3.116 (3)	148
O7—H10···O3^v^	0.85	1.94	2.770 (3)	166
O7—H9···O1^ii^	0.85	2.61	2.992 (2)	109
O7—H9···O2^ii^	0.85	1.85	2.680 (3)	166
O8—H12···N1^iii^	0.85	1.98	2.819 (3)	172
O8—H11···O1^vi^	0.85	1.87	2.713 (2)	175

Symmetry codes: (iii) −*x*, *y*−1/2, −*z*+1; (iv) *x*−1, *y*, *z*; (v) −*x*, *y*+1/2, −*z*+1; (ii) *x*, *y*, *z*−1; (vi) *x*+1, *y*, *z*−1.
